# Combating Obesity through Healthy Eating Behavior: A Call for System Dynamics Optimization

**DOI:** 10.1371/journal.pone.0114135

**Published:** 2014-12-15

**Authors:** Norhaslinda Zainal Abidin, Mustafa Mamat, Brian Dangerfield, Jafri Haji Zulkepli, Md. Azizul Baten, Antoni Wibowo

**Affiliations:** 1 School of Quantitative Sciences, College of Arts and Sciences, Universiti Utara Malaysia, Sintok, Kedah, Malaysia; 2 Department of Mathematics, Faculty of Science and Technology, Universiti Malaysia Terengganu, Kuala Terengganu, Terengganu, Malaysia; 3 Department of Management, School of Economics, Finance and Management, University of Bristol, Bristol, United Kingdom; INIA, Spain

## Abstract

Poor eating behavior has been identified as one of the core contributory factors of the childhood obesity epidemic. The consequences of obesity on numerous aspects of life are thoroughly explored in the existing literature. For instance, evidence shows that obesity is linked to incidences of diseases such as heart disease, type-2 diabetes, and some cancers, as well as psychosocial problems. To respond to the increasing trends in the UK, in 2008 the government set a target to reverse the prevalence of obesity (POB) back to 2000 levels by 2020. This paper will outline the application of system dynamics (SD) optimization to simulate the effect of changes in the eating behavior of British children (aged 2 to 15 years) on weight and obesity. This study also will identify how long it will take to achieve the government’s target. This paper proposed a simulation model called Intervention Childhood Obesity Dynamics (ICOD) by focusing the interrelations between various strands of knowledge in one complex human weight regulation system. The model offers distinct insights into the dynamics by capturing the complex interdependencies from the causal loop and feedback structure, with the intention to better understand how eating behaviors influence children’s weight, body mass index (BMI), and POB measurement. This study proposed a set of equations that are revised from the original (baseline) equations. The new functions are constructed using a RAMP function of linear decrement in portion size and number of meal variables from 2013 until 2020 in order to achieve the 2020 desired target. Findings from the optimization analysis revealed that the 2020 target won’t be achieved until 2026 at the earliest, six years late. Thus, the model suggested that a longer period may be needed to significantly reduce obesity in this population.

## Introduction

Obesity is a term referring to the condition of excess fat [1]. It has been classified as a chronic disease and has become a worldwide noticeable problem, affecting both rich and poor countries. Research indicates that obesity is linked to the incidences of various diseases such as heart disease, type-2 diabetes, some cancers, and numerous psychosocial problems [2]. It is well accepted that obesity in the population is caused by an imbalance, a gap between calories consumed and expended [3]. Over the last two decades, changing in diet propensity with the growing consumption of food away from home contributes to obesity [4]. Food prepared away from home, particularly fast-food, contains higher saturated fat and energy density, and has more added sugar and larger portion sizes ([5], [6]). Public health literature suggests that greater frequency of outside food consumption is associated with weight gain and increased body mass index (BMI) and obesity ([5], [7]). For children, foods containing higher fat, sugar, and cholesterol reduce the IQ in later childhood [8]. In contrast, healthy food options are associated with improved intelligence [9].

Obesity is now no longer rare in children and the prevalence is increasing at an alarming rate. Since obesity is a carryover process and has long-term negative impacts on various life aspects [2], obesity prevention should ideally start in childhood [1]. Statistical data in the UK shows that the prevalence of overweight and obese children in British has increased since 1995 [10]. To respond to the increasing trends in the UK, in 2008 the government set a target to reverse the prevalence of obesity (POB) back to 2000 levels by 2020 [11]. Although some progresses have been initiated to tackle childhood obesity in the UK, the level of obesity still remains high, especially in lower socioeconomic groups [11]. Thus, the cause of the failure must be determined in order to achieve a successful solution. From a quantitative perspective, one of the failures in solving any complex system is inadequate tools to design, analyze, and implement actions and policies [12].

Reviews of the published literature have revealed that a variety of techniques have been employed in the quest for effective obesity amelioration. Most are direct experimental studies such as behavioral change interventions, which are assessed using randomized controlled trials. Their objective is to determine if the change has had an impact on weight reduction but they have not provided sufficient evidences of lasting effects, suggesting that more time is required for the changes to create an effect at the population level ([13], [14]). Alternatively, statistical models have been created but they mostly look at the prediction of trends and normally need an abundance of historical data. Feedback processes are evident in the system when viewed holistically but these are not clearly defined in the statistical or analytical models.

One possible way to overcome the shortcomings of the above methods is the use of the system dynamics (SD) model. A model based on the SD methodology is different from other obesity models because it can re-create the obesity process at the population level with all relevant variables considered endogenously and with the feedback processes, where evidences are properly represented [15]. Moreover, non-numerical (soft) variables can be included in the model. SD concentrates on trends in behavior and de-emphasizes a fit to particular data points. One of the important usages of SD is to evaluate policies for system improvement [16]. SD serves as a medium to test various policy improvements under a wide range of performances. Optimization is one of the testing tools and a very limited number of studies was found to analyze the behavior of a system under changing policies using optimization ([17], [18]). Thus, the use of optimization in policy evaluation is still considered novel in the SD area. For example, although SD optimization has been used to solve numerous policy solutions (see [19], [20], [21]), none of the SD studies, to the best of the researchers’ knowledge, have paid attention to the behavior solution related to obesity.

### Aim of the Analysis

This study considers the UK government’s target to remove obesity as a public health concern by 2020**.** As part of the overall research objectives, this study attempts to demonstrate the contribution offered by SD modeling in an important area of public health. The model is applied as an experimentation tool to attain useful insights into the effects of eating behavioral change on the measurement changes. The outcomes of changes are examined within the following trends: average weight (AW), average body mass index (ABMI), and the POB. This study considers how eating behavior modifications in the British child population might lead to the successful POB target by 2020. This study aims to answer the following research questions: first, whether the target set in 2000 will be accomplished by 2020, and second, if not, how long will it take to achieve this measurement target?

### Structure of the Paper

The rest of the paper is organized as follows: The next section presents a broad sample of studies from the SD literature related to various obesity issues. In the methodology section, the conceptual framework on which the model is based on is defined. Next, SD model of obesity behavior is formulated by highlighting the causal relationship and feedback dynamics. Then, these dynamic relationshiops are integrated into a running model that is being validated. The final section summarises the conclusion and offers recommendations for future works.

## Analytical Framework of the Study

### Overview of System Dynamics Optimization

Optimization is defined as achieving the best solution from the set of available alternatives with regard to some criteria [22]. From the SD point of view, optimization is used to improve the model results via model performance (policy optimization) or to fit the model to available time-series data (calibration optimization) [23]. The choice between the two types of optimization depends on the model purpose. Normally, SD researchers and practitioners depend on their intuitions, experiences, and trial and error approach for policy design and improvement where the policies are tested and modified and the process is repeated until satisfactory results are found. These approaches provide a good direction for policy improvement but are time consuming, especially for beginners and for those who work with insufficient computer facilities ([24], [20]). The situation is now changing and more efforts have been extended with the development of the policy design methods. There are two types of policy design methods, an application of control theory method and simulation by optimization. The latter has emerged recently as a result of the advanced development of computer software.

### System Dynamics Studies in Tackling Obesity

Several obesity studies that use the SD approach have been published, including the work of Abdel-Hamid [25], who used the SD approach to model body metabolism and energy regulation. Abdel-Hamid assessed the factors of dietary intake and exercise and how these components interact to determine adult body weight. In 2004, Homer and colleagues [26] modeled the impact of the caloric imbalance on the changes in body weight and BMI of the adult population in the USA. In Homer et al. [27], the aim was to model BMI trends of various age categories in the population. However, according to the researchers’ knowledge, none of these studies used optimization to find a solution to obesity. To fill this gap, the hybrid of two approaches of calibration and policy optimization is adopted for the current study. The desired AW target is entered as a future time-series and the model is optimized to this series. The objective is to alter the trend so that by 2020, the POB is back to what it was in 2000. Finally, in contrast with the above SD models, which focus either on the individual or the entire US population, the focus here is on the British child population aged 2–15 years, segregated by gender and three age bands.

## System Dynamics Methodology

### Overview of System Dynamics

SD was introduced in the late 1950s and Professor Jay Forrester is the trailblazer of the methodology [28]. It is a computer-aided approach to studying the behavior of a complex system over time. SD modeling initially involved mapping, then evolved with the model’s development, which uses a software package, for instance, Vensim, Stella, iThink, or Powersim. In terms of usage, it is an appropriate method to tackle strategic level issues related to policy decisions. Specifically, SD employs a model to test policies and to overcome policy resistance. SD is different from other modeling approaches as it deals with the complex problem which contains the ***feedback loops***, non-linear and time ***delays*** that affect the behavior of the entire system [16]. Due to its capability, the applications of SD in various health studies are well reported in literatures such as obesity [29], [30] and Chlamydia screening [31].

### Data Sources

This study focused on the British child population, aged between 2 and 15 years, as an overall approach to prevent obesity at the population level. The data used in this study were obtained from the Health Survey for England (HSE) [10] as well as other published sources.

### Model Description

**Figure 1:**
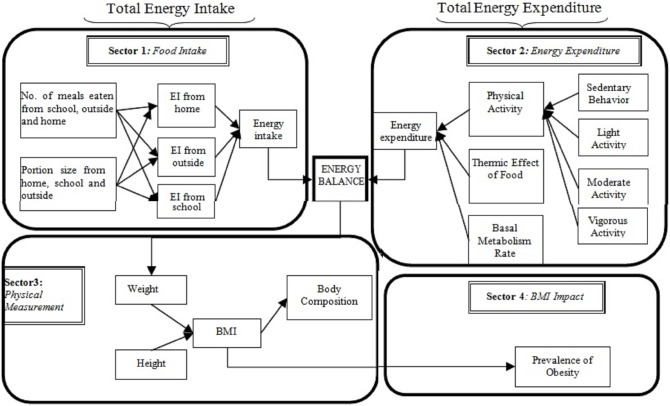
The interaction of eating and physical activity behavior on weight and obesity; Here, EI = energy intake, BMI = body mass index.

Fig. 1 demonstrates the framework to depict the structural assumptions of obesity process. The diagram allows the integration of four different sectors of food intake, energy expenditure (EE), physical measurement, and BMI impact into a single diagram. All these sectors have a specific function when considering that the ultimate purpose of the model is to evaluate the effects of changes in eating behavior on weight and obesity.

**Figure 2:**
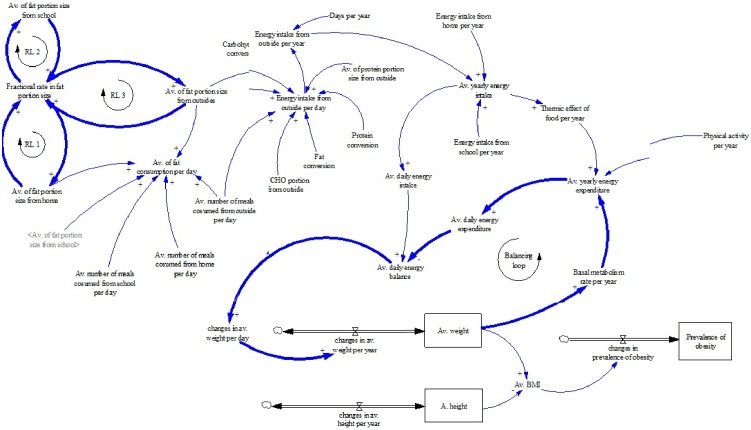
Causal loop diagram of the interaction of eating and physical activity behavior on weight and obesity.

Fig. 2 is an extension of the sub-sector diagram, designed to describe the obesity process from feedback explanations. The diagram was designed using the causal and loop (CL) tool, which consists of variables connected by positive (+) or negative (–) links. A positive link between variables A and B means an increase in A will result in an increase in B. Meanwhile, a negative link means that if the A variable increase, the B variable will decrease in opposite direction [16]. In regards to food consumption, the greater portion size and number of meals taken result in a larger consumption of total fat, whereas the opposite condition applies for lower consumption. Average fat portion size either from home, school, or outside meals is driven by a parameter called fractional rate. Changes in fractional rate result in changes in the average fat portion size, and this influencing process is explained from the three reinforcing loops of RL1, RL2 and RL3.

The overall obesity mechanism from energy balance (EB) and its weight and basal metabolism rate (BMR) progress were described and marked with the BL symbol [29]. EB is the difference between energy intake (EI) and energy expenditure (EE). Thus, changes in any of these energies affect changes in EB. A daily positive EB is stored in muscle or fat. An increase in weight itself causes higher EE, specifically energy from BMR. An increase in BMR energy causes increased EE in total, which in turn tends to bring EB back to zero. This BL causes weight to reach a new EB value in the long run. Equivalence between EI and EE ensures no weight change. The entire process can take years to show up as changes in the AW of the population [26]. The model is calibrated in years and so changes in daily EB and weight variability are smoothed out to result in a less sharp AW change in a year. However, because eating and performing physical activity (PA) is a daily process, the model additionally computes EI and EE in daily units to aid understanding and to reflect common health nomenclature. Weight and BMI are measured using the formula of 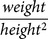 [32]. The more weight an individual has, the higher the BMI value, and the opposite condition for weight loss.

### Model Development

This paper developed a simulation model called Intervention Childhood Obesity Dynamics (ICOD) by focusing the interrelations between various strands of knowledge in one complex human weight regulation system. The ICOD obesity model was successfully developed using the Vensim software for formulating, analyzing, and experimenting purposes [33]. In this study, the model was simulated from 1970 to 2030, and the output divided into Phase 1 (1970–2012) and Phase 2 (2013–2030). Phase 1 represents the past and present situations that lead to obesity, whilst Phase 2 refers to the capability of the ICOD model to reverse the future of AW, ABMI, and POB trends. The data were collected from publishing sources [10] and a literature review [25], [26], in order to simulate the model. The time step for the simulation is 0.0625.

### System Dynamics Optimization Process

Overall, there are five steps in the optimization process [34]. As the first step, the important concepts need to be determined before the optimization process begins. These concepts are payoff function and weight. The payoff function is a formula which expresses the objective function. Next, the weight value must be assigned to the payoff function and the value should always be 1.0 for a calibration optimization [23]. In calibration optimization, Vensim takes the difference between the model variable and the data value, multiplies it by weight, squares it and adds it to the error sum, which is minimized. Then the parameters are considered. The optimization process continues by searching for the best solution that best fits the simulated AW to the time series data entered as desired AW. The optimization process occurs by maximizing the payoff function. The value of the payoff function is normally negative and after optimization the value should be less negative. The best payoff value after optimization would be zero [23].

### The Adoption of System Dynamics Optimization

**Figure 3:**
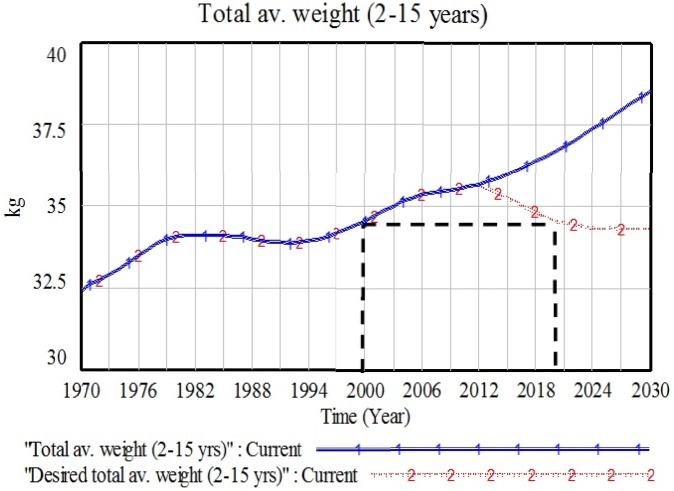
Base case trajectory for total average weight (blue line) and a plausible desired total average weight (red dotted line).

Table 1 presents a plausible AW target needed by 2020 and Fig. 3 illustrates a graph for weight changes from the baseline, starting in 2013, that would be compatible with UK government policy. We decided on weight because for the nation to reverse the rising tide of population obesity, everyone needs to be able to reach a healthy weight. With that, the achievement in POB only will be achieved [11]. The upper (blue) line in Fig. 3 shows the simulated trend (base case) for total AW whereas the lower (red) line is a desired weight trajectory which passes through the target needed by 2020 (≈34.6 kg). Total AW is the weight measured from the total of six model categories of three age groups and genders. The trend is presented from a total population (2–15 years) as a broader strategy to tackle population obesity. In order to achieve the desired weight target of 34.55 kg in 2020, the changes must be made in single or combination of parameters. In our study, we choose the year 2013 as our initial year for the policy changes to be rolled out.

**Table 1:** Changes in average weight values from the past (1970–2010) and into the future (2020–2030).

Average weight (kg)	1970	1980	1990	2000	2010	2020	2030
Total average weight (Baseline)	32.40	34.06	33.91	34.55	35.55	36.69	38.56
Desired total average weight						34.55	34.31

Due to the inclusion of a future data series by this analysis, the overall process is effectively a hybrid of policy and calibration optimizations. Using optimization, the model searches and chooses the best parameter value to fit with a given future weight series data, ranging from 2013 to 2030. In response to eating behavior changes, public policy changes cannot produce a sudden step response, so we have optimized the rate of change parameters which are assumed to change in a gradual linear fashion over a period of years. Obviously the trajectory could be different with a slower initial decrease and then a sharper fall to the target value in 2020. This is managed in the model using the RAMP function, one of the functions available in Vensim software. A RAMP function is a flow that is increasing or decreasing linearly and not constant over time. Prior to this experiment, the portion size and number of meals variables were reduced linearly from 2013 until 2020 using RAMP function. We have not yet explored other possible trajectories.

To achieve the necessary reduction in AW by 2020, the optimization process was performed at rates of change in eating variables. It is reflected in the structure of equation 1 below. This generalized can be applied to eating parameters, although the sign needs to reflect whether a reduction or an increase is desirable as shown by the ± sign in equation 1. Changes in behavior are assumed to commence in 2013. Due to a delay between the behavior changes and their effect, obviously no results have been observed in the beginning of the intervention period, especially in 2013. The impact will only be seen after at least a year.
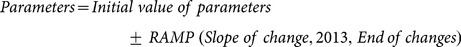
(1)


**Figure 4:**
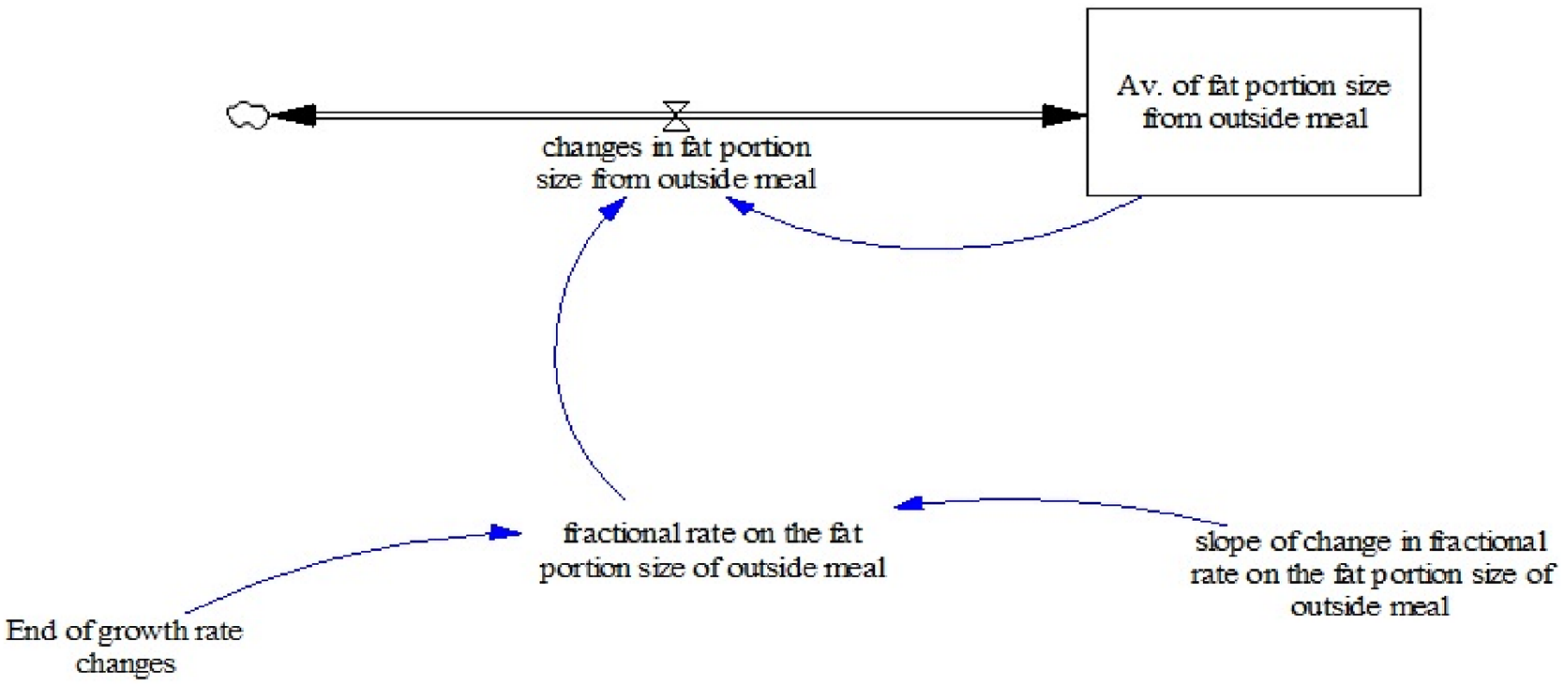
Structure for modeling the average of fat portion size from outside.

In the optimization experiment, changes are made in the selected items of eating parameter(s) while all PA parameters are held as they were in the base case. However, only two parameters were chosen for the experiments, which are fractional rate on the fat portion size of outside meal (called A) and fractional rate in total meals consumed (called B). Parameter A represents the proportion of portion size of outside meals eaten per year, while B parameter represents the proportion of the number of meals eaten from home, school, and outside meals per year. Alterations made in eating parameters work by decreasing the amount of EI and total EE. Changes in both EI and EE result in changes in EB, where greater reduction in EB leads to more weight reduced [30]. To illustrate how the parameter was modified during optimization, consider an example of parameter A and its modeling structure presented in Fig. 4. The new equation 2 using the RAMP function (see eq.4) is introduced for immediate changes response in parameter A. Both original (eq.3) and revised (eq.4) equations applied during the experiment are presented as well. The slope range for the new parameter A is between 0.0005–1 and changes in parameter A between the range values. The similar modeling structure was applied to parameter B.
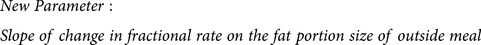
(2)

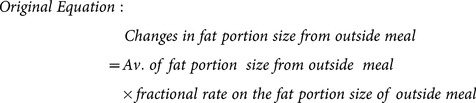
(3)

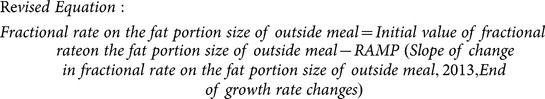
(4)


**Figure 5:**
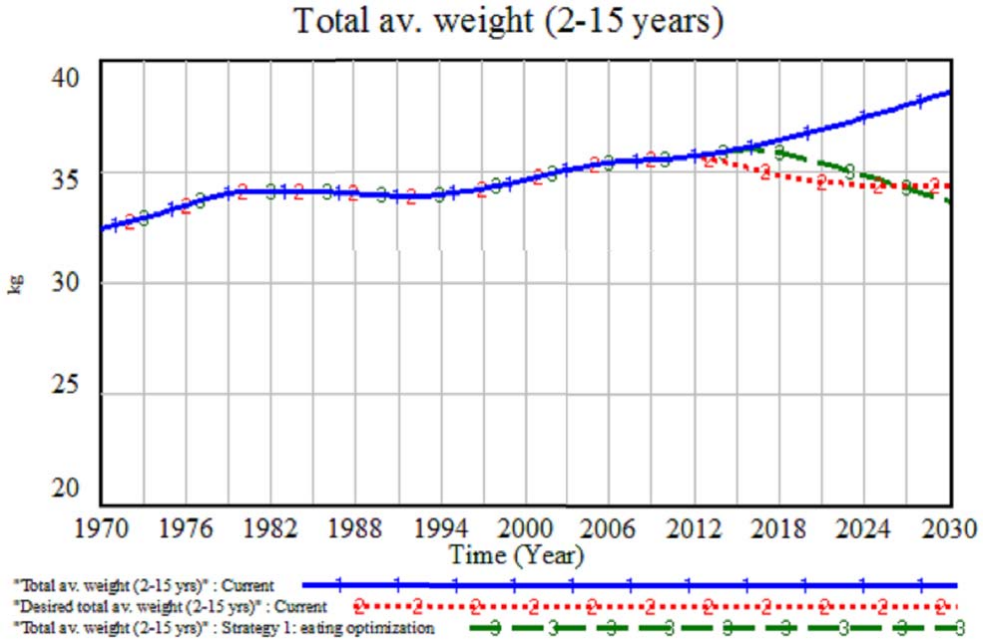
Comparison of eating optimization (green line) with the desired target (red line) for the average weight projections between 2013 and 2030.

During optimization, the original parameter becomes a variable as the search routine works to achieve the best objective function result. Through this change, the closest result (green line) to the red line for AW as presented in Fig. 5 is achieved. Using optimization, the model provides a suggestion on the best reduction value in the parameters to fit with future weight target data. The solution suggested from optimization depends on the number of parameters and time duration factors. Optimization experiments possibly can be experimented on more than two parameters at a time and the searching process will be more difficult and time-consuming if involved with a number of parameters [23]. Using SD optimization, suggestion for the solution might not offer the exact point-by-point trend to fit into a given data series, but the closest solution to the desired target trends of simulation periods.

### Model Validation

**Figure 6:**
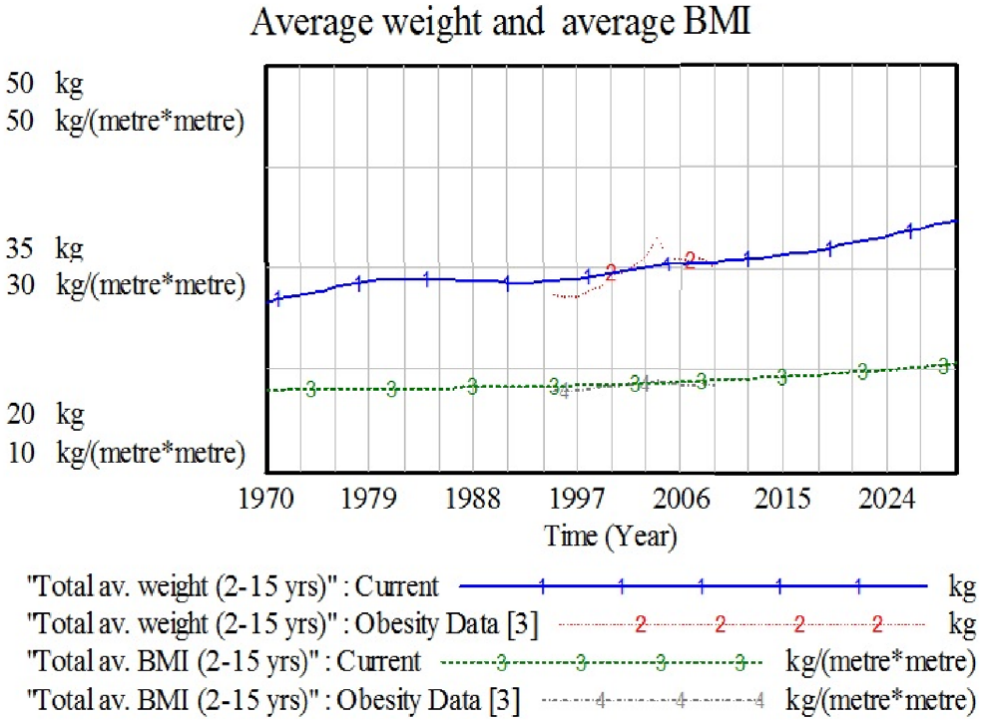
Fitting trends between real data and simulated trends of weight and BMI.

**Figure 7:**
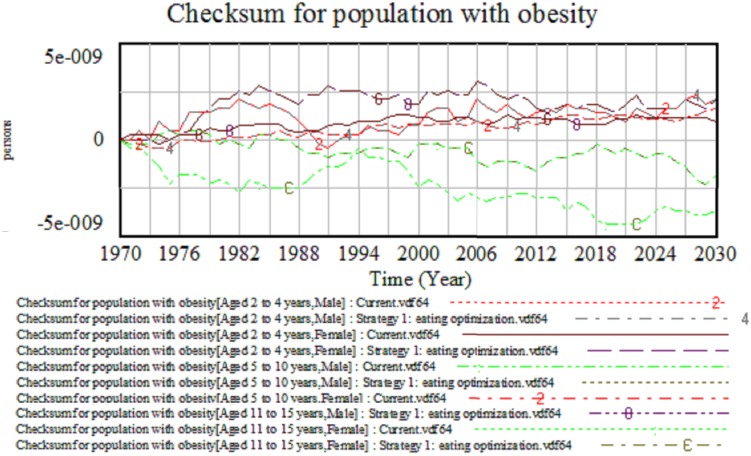
Balanced output for the obese and non-obese population.

To validate the model, the test is done to both structure and behavior of the model as suggested by the Forrester and Senge [35] and Sterman [16]. The ICOD model has successfully passed on behavior reproduction since the real data fit with the simulated trends for both AW and ABMI, as shown in Fig. 6. The other test is mass balance, also known as the checksum test, which does not involve any simulation [30]. This test works to balance the input and output values in the model by identifying whether people (resources) are added to or leak from the model during the course of a simulation. For the balance to be satisfied in this condition, the formula of total population of obese and non-obese must be equal with the whole population. The value for checksum must be equal to zero at all times. Fig. 7 proved that the model has passed the checksum test with the checksum trends fluctuating around zero, allowing for computational accuracy. If the input and output do not balance, then the graph will exhibit a non-zero behavior pattern.

## Results and Discussions

The simulated result from the base case (Current) for total AW is presented in the blue line trend in Fig. 5. Including the optimized values caused a reduction in the average of fat portion size from outside, and the average of annual number of meals. For instance, result from simulation analysis depicts a decrease in meal consumptions in year 2030. Prior to the baseline simulation, meal consumptions are recorded at approximately 35 grams per meals and 1600 meals per year. However, these consumptions have been reduced to 7 grams per meal and almost 250 meals per year after the optimization experiment (see [30]). Although the results from eating optimization (Strategy 1) look unrealistic and are involved with quite drastic changes to achieve the desired weight target, it is important to highlight that the tight timeframe for the target to be achieved is likely to be a major contributing factor. It supports the view that a much longer time frame will be necessary to achieve a reduction in AW at the population level because the time taken to gain weight is known to be shorter than the time taken to reduce it. Apart from that, only two parameters were tested in the model. The results are compressed within these two parameters for the best result achieved.

Table 2 compared the changes in AW, ABMI, and POB measurements before and after optimization experiments. The induced changes in A and B parameters, while PA is maintained as in the base case, will clearly result in a reduction in total EI. However, as Fig. 5 illustrates, the impact on AW is insufficient to achieve the target of 34.55 kg by 2020. Specifically, total AW is gradually decreasing in value but will not get below the target for 2020 until 2026. Total AW in 2020 is reduced to 35.6 kg from a baseline value of 36.69 kg. However, total ABMI will be reduced to 19.1 kg/m^2^ in 2020 and the POB will also exhibit a reduced value of 21.06% in 2020 after an experiment. It is noted that an optimization experiment that reveals both total AW target and the associated POB of 15.43% by 2020 can, in fact, only be achieved by 2026 (see black line in Fig. 5).

**Table 2:** Comparisons of the average weight (AW), average BMI (ABMI), and prevalence of obesity (POB) changes resulting from the optimization experiment.

	AW (kg)		ABMI (kg/m^2^)		POB (%)	
	Baseline	Optimization	Baseline	Optimization	Baseline	Optimization
BASELINE & OPTIMIZATIONEXPERIMENT	2020	2030	2020	2030	2020	2030
	BASELINE (CURRENT)					
	36.69	38.56	19.7	20.61	24.04	28.75
	OPTIMIZATION EXPERIMENT (STRATEGY1)					
	35.60	33.76	19.11	18.02	21.06	12.21

Three things have arisen from this study. The primary findings revealed that the target can only been achieved by 2026, which is six years after the target period. In conjunction with this, we express the caveat here that all these results derive from a limited set of parameter combinations with a limited time duration, which we have experimented with in the model. Since no other research was found that relates to the achievement target, we cannot compare findings of this study with others. However, the work of Stamatakis et al. [36] supports our finding that an increase in future obesity trends in the UK is projected, including in 2020. The other potential solution is through expanding the target beyond 2020. The failure in the achievement target is because the UK government is apt to set targets without adequate prior assessment, and not just in health. Whilst too-low targets can induce complacency, over-ambitious ones are apt to cause ridicule for an administration. The setting of sensible targets cries out for some prior modeling activity, not only to assess the credibility of the target number (usually some years ahead), but also the desired trajectory to be followed in attaining it. The finding also has implications for governments and other bodies who choose to set future health (and indeed other) targets. It is desirable to carry out some modeling in advance to ascertain if the chosen target might just be feasible. Often it seems that the targets are decided on a whim and the consequence of this might be acute embarrassment for the government.

Secondly, this study focuses on the challenges the government faces in order to achieve the desired target. The failure to achieve the target might be due to the ineffectiveness in the policy intervention related to obesity problem. It is not supposed to focus on individual level issues only, such as controlling food consumptions. A more practical approach should encompasses the environmental influence, such as providing the environment that promotes healthier food consumption behavior [37]. Thus, this finding brings an important message to the government and policy makers that the planning strategies for obesity interventions might be better targeted at creating an environment which makes it easier to access healthier food rather than convincing individuals to make healthier choices.

Lastly, this research highlighted that the current UK food policy needs to be reviewed with respect to its effects on children. This is especially the case for school and outside food sources. Most of the teenage population nowadays consumes more outside food than meals at home. The frequent consumption of outside food and sugar-laden drinks has a significant impact on weight gain and obesity [7].

## Conclusions

The ICOD obesity model was successfully developed with the intent to better understand how past eating behavior influences a child’s weight gain and increased obesity. The model is designed to address the issue and build a shared understanding of obesity dynamics in a way that is solidly grounded in the best available science and useful to non-specialists, from policy makers to the public. Using SD calibration and policy optimizations, this study offers insight into how the best of AW, ABMI, and POB reversion values can be achieved in the future direction by controlling portion size and number of meals eaten. Findings from the optimization experiment revealed that the government’s desired target in both AW and POB is unlikely to be achieved by 2020 and the target can be only achieved by 2026. Therefore, the model suggested a longer period may be needed to significantly reduce childhood obesity in this population. This paper concludes that the SD optimization is a meaningful approach to guide the food stakeholder to understand and to experiment on the dynamic situation of obesity from an eating behavior perspective.

## Limitations and Future Works

The ICOD model enables decision makers, practitioners, researchers and the public to learn and quickly access information about the effect of nutrition on the dynamics of obesity. However, as with any model, the ICOD model is not appropriate for all purposes. To address the issue, this study has some limitations that need to be highlighted. In conducting this research, limitations in data acquisition have been experienced. There is a lack of historical national survey data, especially on EI and PA, which limits the scope for model validation. Because of that, the development of the obesity model is derived from the best information available from the literature and also from expert knowledge. For the purpose of simplification, we assumed the model exhibits similar behavior in all age groups and gender categories in terms of eating, PA, and metabolic aspects. In reality, there might be differences in the regulation of EI and EE among the different age groups and genders, not only from quantitative aspects but also a qualitative difference. Another limitation is that RAMP trajectory trends were only experimented with a limited number of variables tested in the optimization experiment.

From this limitation, future research can be done by extending the model. One of the interesting areas for extending the model would be that the heterogeneity issues might be addressed and tested for future research direction. Secondly, if the target to overcome obesity by changing eating behavior is not achievable by the year 2020, we need to consider a few alternative approaches to this issue. One plausible solution to this problem is to focus on multiple aspects of behavioral changes. For instance, the combination of PA and eating behavioral changes might offer the better solution for BMI or weight reduction ([38], [39], [40]). Therefore, our study is striving to explore the dynamic effect of changes in both behavior to the weight and BMI reversing trends. Lastly, we also plan to conduct experiments with an unlimited number of parameters for future research direction from optimization work.
